# Exploring the impact of glutathione *S*-transferase (GST)-based metabolic resistance to insecticide on vector competence of *Anopheles funestus *for *Plasmodium falciparum*

**DOI:** 10.12688/wellcomeopenres.15061.2

**Published:** 2019-10-11

**Authors:** Cyrille Ndo, Edmond Kopya, Helen Irving, Charles Wondji

**Affiliations:** 1 Department of parasitology, Centre for Research in Infectious Disease (CRID), Yaoundé, P.O. Box 13591, Cameroon; 2 Department of Biological Sciences, Faculty of Medicine and Pharmaceutical Sciences, University of Douala, Douala, P.O. Box 24157, Cameroon; 3 Vector Group, Liverpool School of Tropical Medicine, Pembroke Place, Liverpool, L3 5QA, UK; 4 Institut de recherche de Yaoundé (IRY), Organisation de Coordination pour la lutte Contre les Endémies en Afrique Centrale (OCEAC), Yaoundé, P.O. Box 288, Cameroon; 5 Department of Animal Biology and Physiology, Faculty of Sciences, University of Yaoundé I, Yaoundé, P.O. Box 337, Cameroon

**Keywords:** Malaria, Insecticide resistance, Anopheles funestus, Plasmodium falciparum, metabolic resistance, GSTe2

## Abstract

**Background: **Malaria control heavily relies on insecticide-based interventions against mosquito vectors. However, the increasing spread of insecticide resistance is a major threat. The extent to which such resistance, notably metabolic resistance, influences the development of the *Plasmodium* parasite and its impact on overall malaria transmission remains poorly characterized. Here, we investigated whether glutathione S-transferase-based resistance could influence *Plasmodium falciparum* development in *Anopheles funestus*.

**Methods:** *Anopheles funestus* females were infected with *P. falciparum* gametocytes and midguts were dissected at day 7 post infection for detection/quantification of oocysts. Infection parameters were compared between individuals with different L119F-GSTe2 genotypes, and the polymorphism of the GSTe2 gene was analyzed in infected and uninfected mosquito groups.

**Results: **Overall, 403 *An. funestus*  mosquitoes were dissected and genotyped. The frequency of the L119F-GSTe2 resistance allele was significantly higher in non-infected (55.88%) compared to infected (40.99%) mosquitoes (Fisher's exact test, P<0.0001). Prevalence of infection was significantly higher in heterozygous and homozygous susceptible genotypes (P<0.001). However, homozygous resistant and heterozygous mosquitoes exhibited significantly higher infection intensity (P<0.01). No association was observed between the GSTe2 polymorphism and the infection status of mosquitoes.

**Conclusion: **Altogether, these results suggest that GSTe2-based metabolic resistance may affect the vectorial competence of resistant *An. funestus* mosquitoes to *P. falciparum* infection, by possibly increasing its permissiveness to *Plasmodium* infection.

## Introduction

 Intense control efforts have been deployed since 2000 to reduce the burden of malaria in Africa, relying heavily on insecticide-based interventions, including the scale-up of long-lasting insecticide nets (LLINs) and indoor residual spraying (IRS). The proportion of households possessing at least one LLIN has increased from less than 50% in 2010 to an estimated 80% in 2016. Similarly, the proportion of populations at risk of malaria sleeping under LLINs has increased from 24% to 54% in the same time frame ( WHO, 2017). The implementation of these vector control measures led to significant reduction of malaria incidence and mortality by 21% and 31%, respectively on the African continent, between 2011 and 2015 ( WHO, 2017). Unfortunately, the heavy use of insecticides in the public health and agriculture sectors has in turn selected resistance in major vector mosquitoes *An. gambiae*, *An. coluzzii* and *An. funestus* *s.s.* (hereinafter *An. funestus*) across the continent, and this is considered as a serious threat to sustainable malaria control ( Ndo *et al*., 2018; Ranson & Lissenden, 2016). There is a fear that such resistance could impact malaria vector competence and increase malaria transmission. However, little is known on the interactions between resistance and mosquito’s ability to harbour and transmit malaria parasites, preventing us from anticipating the epidemiological impact of insecticide resistance.

 In *Anopheles* mosquitoes, insecticide resistance is driven mainly by two mechanisms: alteration of target sites of insecticides and metabolic resistance through an over-expression of detoxification genes ( Corbel *et al*., 2007; Liu, 2015; Menze *et al*., 2016). The target-site insensitivity resistance is the best characterized and can be easily monitored using various diagnostics ( Bass *et al*., 2007; Martinez-Torres *et al*., 1998). Point mutations in the gene coding for the voltage-gated sodium channel confer cross resistance to dichlorodiphenyltrichloroethane (DDT) and pyrethroids insecticides named for knockdown resistance (kdr) ( Ranson *et al*., 2000), while mutations in the *ace-1* gene, which encodes the acetylcholinesterase enzyme, confer cross-resistance to carbamate and organophosphate insecticides ( Alout & Weill, 2008; Weill *et al*., 2004). Contrary to the target site mechanism, monitoring of metabolic resistance is more complex and requires advanced genomic analytical methods such as qPCR, microarrays or RNA sequencing. Metabolic mechanisms are the result of over-expression, either by amplification and/or upregulation of detoxification genes (Cytochrome P450s, glutathione S-transferases and esterases) ( Hemingway & Ranson, 2000).

 The successful management of insecticide resistance will require a good understanding of the mechanisms involved, but more importantly, its impact on vectorial capacity/competence and malaria transmission. The selection of insecticide resistance in mosquitoes is thought to interfere with the pathogens they transmit during one of the main steps of development, including parasite differentiation, proliferation, and migration to the specialised tissues. For example, enzymatic modifications involved in metabolic resistance could render mosquito internal environment toxic for the parasite or may influence one or many steps of the immune response, from the recognition of the parasite as foreign, to the deployment of the killing mechanism ( Rivero *et al*., 2010).

Despite the widespread distribution of resistance, its impact on the ability of *Anopheles* vectors to transmit malaria and therefore, its epidemiological impact remains unclear. This is particularly true for metabolic resistance mechanisms since no molecular marker was previously available to assess its impact contrary to target-site resistance [e.g. knockdown resistance ( *kdr*)] for which DNA-based diagnostic tools were designed two decades ago ( Martinez-Torres *et al.*, 1998). Progress made recently in *An. funestus* has led to the detection of a DNA-based marker for the glutathione S-transferase epsilon 2 gene ( *GSTe2*) consisting of one single amino acid change (L119F) in an upregulated *GSTe2* ( Riveron *et al.*, 2014). Geographical distribution of this point mutation strongly correlated with insecticide resistance patterns across Africa. Functional characterization of recombinant *GSTe2* further supported the resistant allele as being more efficient at metabolizing insecticide, notably DDT, by enlarging the GSTe2-DDT biding cavity, leading to increased access and metabolism of the insecticide ( Riveron *et al.*, 2014). Taking advantage of availability of this new DNA-based *GSTe2* marker, we investigated the impact of a GST-mediated metabolic resistance on the vector competence of the major malaria vector *An. funestus*. We showed that the L119F-GSTe2 mutation conferring pyrethroid/DDT resistance could influence *P. falciparum* infection in field populations of this vector.

## Methods

### Study sites

 Mosquitoes originated from Mibellon (6 ° 46'N, 11 ° 70'E) and Obout (3° 7'N, 11 ° 65'N) situated 350 km and 25 km, respectively, from Yaoundé the capital city of Cameroon. Mibellon is situated in the Adamaoua region in the humid savannah zone. The climate is Sudano-Guinean characterized by an eight-months rainy season from March to October, and a dry season of four months extending from November to February ( Olivry, 1986). Two main malaria vector species namely *An. gambiae sl* and *An. funestus* are routinely found in the village, with the latter being the most abundant throughout the year. Both vectors have developed high levels of resistance to pyrethroids (deltamethrin and permethrin) and organochlorides (DDT) and moderate resistance to carbamate (bendiocarb) ( Menze *et al*., 2018). *Anopheles funestus* was found to actively transmit *Plasmodium* parasite in the locality, with an infection rate of up to 3.7% ( Menze *et al*., 2018; Ndo *et al*., 2016).

Obout is located within the dense rainforest area of the Centre region (Southern Cameroon). The climate is similar to that of Equatorial Guinea, characterized by two rainy seasons extending from August to October, and from April to June. There are also two dry seasons running from November to April, and from June to July ( Olivry, 1986). *Anopheles gambiae sl* and *An. funestus* are the main vector species found in the village ( Ndo *et al.*, 2018). High *Plasmodium* infection rates reaching 23% were reported in these species which have also developed resistance to DDT and pyrethroids, notably deltamethrin and permethrin ( Ndo *et al*., 2018).

### Mosquito sampling and identification

Mosquitoes were collected between 8–11am inside human dwellings using electric aspirators (Rule In-Line Blowers, Model 240). They were brought back to the insectary where initial species identification was performed based on morphological criteria ( Gillies & Coetzee, 1987). They were later confirmed as *An. funestus* using a PCR assay ( Koekemoer *et al*., 2002). All blood-engorged *An. funestus* mosquitoes were kept four days in cages until eggs were mature. Gravid females were allowed to oviposit individually using a forced egg-laying method ( Morgan *et al*., 2010). Progenies were pooled and reared to adulthood under standardised conditions.

### Experimental infections

A total of 20 infection experiments were conducted, each using blood from different gametocyte carriers, with different parasite density. Gametocytes of *P. falciparum* were collected from the blood of infected children at local primary schools of the locality of Okola (Centre, Cameroon) as previously described ( Ndo *et al*., 2016). Briefly, presence of different parasite stages in the blood was detected by examining thick blood smears stained with 10% Giemsa under light microscope (Leica DM 300). The number of gametocytes was counted against 500 leucocytes, and an estimation of its density in the blood was done based on an average of 8000 white blood cells /µl.

Blood was collected from selected gametocyte carriers by venepuncture into heparinized tubes. It was immediately centrifuged for 5 minutes at 2000 RPM using a centrifuge (Model EBA 20, Hettich Lab Technology) placed inside an incubator (Jouan EB115) set at 37°C, and the donor's plasma was replaced by the same volume of European AB malaria-naïve plasma (Catalogue number H4522, Sigma-Aldrich, Taufkirchen, Germany). Three to five day old F1 female mosquitoes were allowed to feed through an artificial parafilm membrane maintained at 37°C using a circulating heating water bath (Fisher Scientific INC, Isotemp 4500H5P, Pittsburgh USA). After 45 min, fed mosquitoes were sorted and placed in separated cups until dissection of midguts at day 7 (D7) post-infection. Dissected midguts were stained with 0.4% mercurochrome before they were examined under light microscopy (Leica, Model DM 300) at objective 40X for detection and quantification of oocysts. All carcasses were preserved at -20°C until DNA extraction was performed.

Throughout the experiments, we used the *An. coluzzii* Ngousso strain as control sample to monitor for the effectiveness of blood handling procedure and infectivity of gametocytes, since this strain is well adapted to feed on artificial parafilm membrane and is known to be highly susceptible to *P. falciparum* infection ( Ndo *et al*., 2016). The *An. coluzzii* Ngousso strain originated from Yaoundé (Cameroon) and is routinely maintained at the insectary of the Organisation de Coordination pour la lutte Contre les Endémies en Afrique Centrale (OCEAC, Cameroon) since 2006.

### L119F-GSTe2 mutation genotyping

 Genomic DNA was extracted using Livak's method ( Livak, 1984) from carcasses of infected and non-infected mosquitoes after dissection of midguts. Genotypes of L119F-GSTe2 mutation were determined after DNA amplification using allele specific PCR diagnostic assays using two outer and two inner primers ( Tchouakui *et al*., 2018). Details of the sequences of primer used are presented in Table 1. PCR was performed in Gene Touch thermalcycler (Model TC-E-48DA), in a reaction volume of 15 μl using 10 µM of each primer, 10X Kapa Taq buffer A, 0.2 mM dNTPs, 1.5 mM MgCl _2_, 1U Kapa Taq (Kapa biosystems 5U/µl, Cat: 07958471001) and 1µl of genomic DNA as template. The initial denaturation step at 95°C for 2 min was followed by 30 cycles of 94°C for 30 s, 58°C for 30 s, 72°C for 1 min and a final extension step at 72°C for 10 min. PCR products were separated on 2% agarose gel allowing clear discrimination of the genotypes. The size of the diagnostic band was 523 bp for homozygous resistant (RR) and 312 bp for homozygous susceptible (RS), while heterozygous (SS) showed the two bands.

**Table 1.** Details of primer sequences used to genotype *L119F GSTe2* mutation in *Anopheles funestus*.

Primers	Sequence (5’ to 3’)
Ndel_Gste2F	GGAATTCCATATGACCAAGCTAGTTCTGTACACGCT
Xbal_Gste2 R	TCTACATCAAGCTTTAGCATTTTCCTCCTT
L119F-Res	CGGGAATGTCCGATTTTCCGTAGAA **t**A **A**
L119-F-Sus	CATTTCTTATTCTCATTTACAGGAGCGTA **a**T **C**

### *GSTe2* gene sequencing

 A total of 30 DNA samples including 15 of infected and 15 of non-infected mosquitoes were randomly selected for sequencing of the *GSTe2* gene using the following primers: Gste2F, 5’GGA ATT CCA TAT GAC CAA GCT AGT TCT GTA CAC GCT 3’ and Gste2R, 5’ TCT AGA TCA AGC TTT AGC ATT TTC CTC CTT 3’ (Eurogentec, Liège Science Park, Belgium). DNA was amplified in a total volume of 15 μl containing 10 µM of each primer (forward and reverse), 10 mM dNTPs, ddH _2_O, 10X buffer A, 1.5 mM MgCl _2_ and 1ul of Kapa Taq polymerase (KapaBiosystems, Cat: 07958471001). PCR conditions were an initial denaturation step of 5 min at 95°C, followed by 30 cycles of 94°C for 30 sec, 58°C for 30 sec and 72°C for 1 min, with final extension at 72°C for 10 min. The size of amplicons was checked after visualization of DNA bands on a 2% agarose gel stained with GelRed nucleic acid dye (Biotium, Cat: 41003) (see Underlying data ( Ndo, 2019)). PCR products were purified using ExoSap PCR Purification Kit (Thermo Fisher Scientific, Waltham, MA, USA; Cat:78201.1.ML) following manufacturer’s instructions, and was sequenced using the forward and reverse primers.

### Data analysis

Parameters analyzed for each infection experiment were the prevalence of infection, as the proportion of mosquitoes infected after midgut dissection, and the infection intensity by calculation of arithmetic mean and median number of oocysts in the midguts of infected mosquitoes.

The geographical distribution of L119F-GSTe2 mutation was assessed by determining allelic and genotypic frequencies in each study sites. The impact of L119F-GSTe2 mutation on vector competence was investigated by comparing the frequency of the L119F-GSTe2 resistant allele in infected and non-infected mosquitoes, and by comparing infection parameters (Prevalence of infection, mean, median oocyst load) between mosquitoes of different genotypes (RR, RS and SS). Prevalence of infection, mean, and median oocyst load were computed and compared using the Fisher's exact test and Mann-Withney test, respectively. P-values less than 0.05 were considered as statistically significant. The software GraphPad Prism v 7.05 was used for all statistical analysis.

Genomic sequence analysis started with systematic detection and correction of base-calling and/or sequencing errors using Bioedit V.7.2.5, after visual inspection of DNA sequence chromatograms. A consensus sequence for each single mosquito was generated using both forward and reverse sequences which were used for analysis of polymorphisms and phylogenetics. Sequences were aligned using MEGA V.6.06 and DNA polymorphism parameters were generated in dnaSP V.5.10. Haplotype networks and maximum likelihood phylogenetic tree were constructed using TCS V.1.21.

### Ethical statements

The study was approved by the Cameroonian Ethical Committee for Research in Human Health (Statement N°2016/10/817/CE/CNERSH). The gametocyte carriers used in this study were enrolled as volunteers. Their parents or legal guardians signed a written informed consent form after the procedures of the study were fully explained to them. All children found infected with the malaria parasites received free antimalarial treatment.

## Results

### Mosquito species identification

 In Obout, 615 *Anopheles* mosquitoes were collected during the study period. According to morphological identification, 91.38% belonged to the *An. funestus* group while the remaining mosquitoes were all *An. gambiae sl* (8.62%). In Mibellon, *An. funestus* was also the main vector species representing 94.92% of the 670 *Anopheles* mosquitoes collected while *An. gambiae sl* represented the rest (5.08%). The molecular species identification of the *An. funestus* group showed presence of *An. funestus s.s.* (97.38%) and *An. leesoni* (2.62%) in Obout, while only *An. funestus s.s.* (hereafter called *An. funestus*) was present in Mibellon.

### *Anopheles funestus* infection

Overall, the blood feeding rate of *An. funestus* through the artificial parafilm membrane was low compared to that of the *An. coluzzii* Ngousso strain used as control (80.42% - 100%) and did not exceed 40% in all the cases (min - max: 1% - 38%). *Anopheles funestus* of both sites showed high susceptibility to natural *P. falciparum* isolates with 72.73% (8/11) and 77.78% (7/9) experiments yielding at least one infected mosquito in Obout and Mibellon, respectively. The overall infection rate was 69.73% Mibellon and 42.74% in Obout. By contrast infection intensity represented by mean and median oocyst load in midgut was moderate in Obout (mean: 7.44±1.20; median: 4) and low in Mibellon (mean: 2.88±0.18; median: 2). ( Table 2).

**Table 2.** Infection parameters in *An. funestus* from Obout and Mibellon.

Sites	Exp	Dissected	Infected	Infection rate (%)	Total oocyst	Mean oocyst	Median oocyst	Oocyst range
**Obout**	N°1	58	38	65.52	177	4.66±0.55	4	1 – 16
	N°2	61	17	27.87	39	2.29±0.41	2	1 – 8
	N°3	22	5	22.73	13	2.6±1.03	1	1– 6
	N°4	27	22	81.48	449	20.41±4.71	13.5	1 – 92
	N°5	22	10	45.45	64	6.4±1.94	6	1 – 21
	N°6	28	12	42.86	45	3.75±0.74	3	1 – 8
	N°7	24	1	4.17	1	1	1	1
	N°8	6	1	16.67	1	1	1	1
	ALL	248	104	41.93	787	7.57±1.22	4	1–92
**Mibellon**	N°9	9	4	44.44	13	3.25±0.63	3	2–5
	N°10	31	17	54.84	27	1.59±0.21	1	1–4
	N°11	14	5	42.86	12	2.4±0.4	3	1–3
	N°12	53	32	60.38	73	2.28±0.20	2	1–6
	N°13	7	7	100	31	4.43±0.89	4	1–8
	N°14	58	52	89.65	181	3.48±0.34	3	1–12
	N°15	13	12	100	35	2.92±0.62	2	1–8
	ALL	185	129	69.73	372	2.88±0.18	2	1–12

Gametocyte density is expressed as number of gametocytes per µl of blood assuming an average of 8000 white cells/µl. Exp: experiment; N°: number.

### Distribution of L119F-GSTe2 resistance allele and *P. falciparum* infection

**Figure 1.**
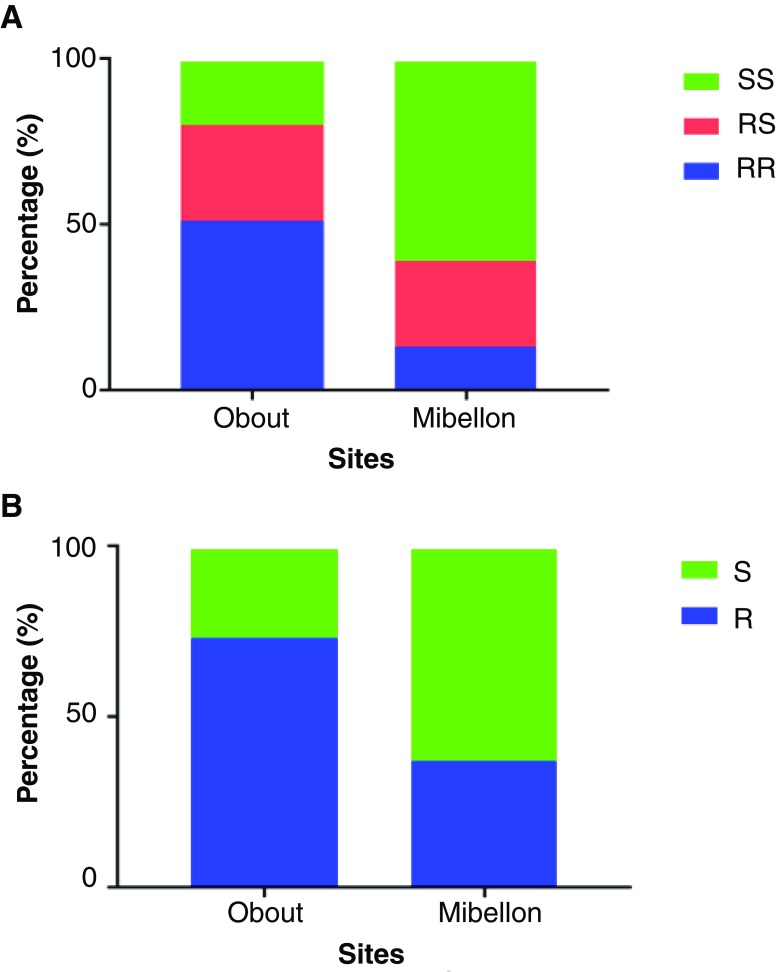
Distribution of L119F-GSTe2 resistance genotypes (A) and alleles (B) in Obout (N:128) and Mibellon (N:185). (SS: homozygous susceptible genotype, RS: heterozygous genotype, RR: homozygous resistant genotype, R: resistant allele, S: susceptible allele).

**Figure 2.**
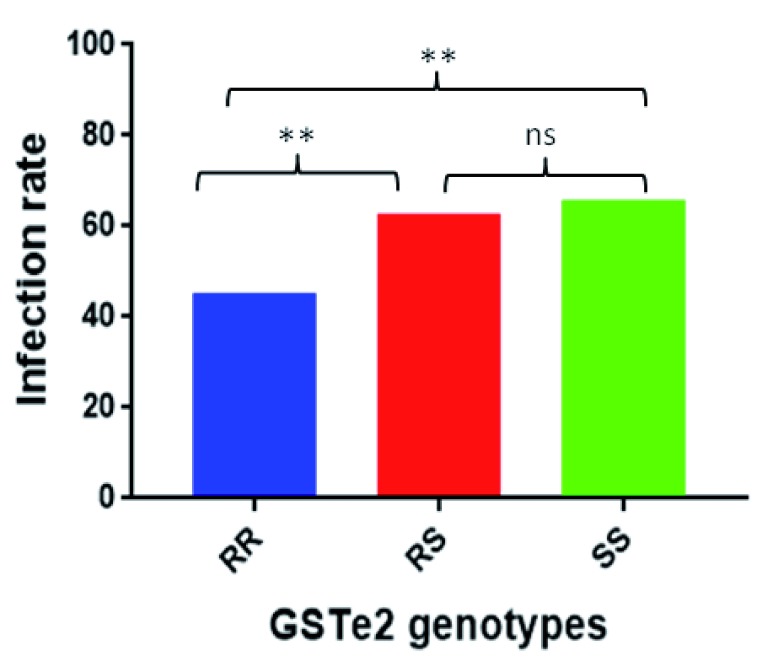
Prevalence of infection according to L119F-GSTe2 genotypes. **: P<0.001; ns: not significant. (SS: homozygous susceptible genotype, RS: heterozygous genotype, RR: homozygous resistant genotype).

**Figure 3.**
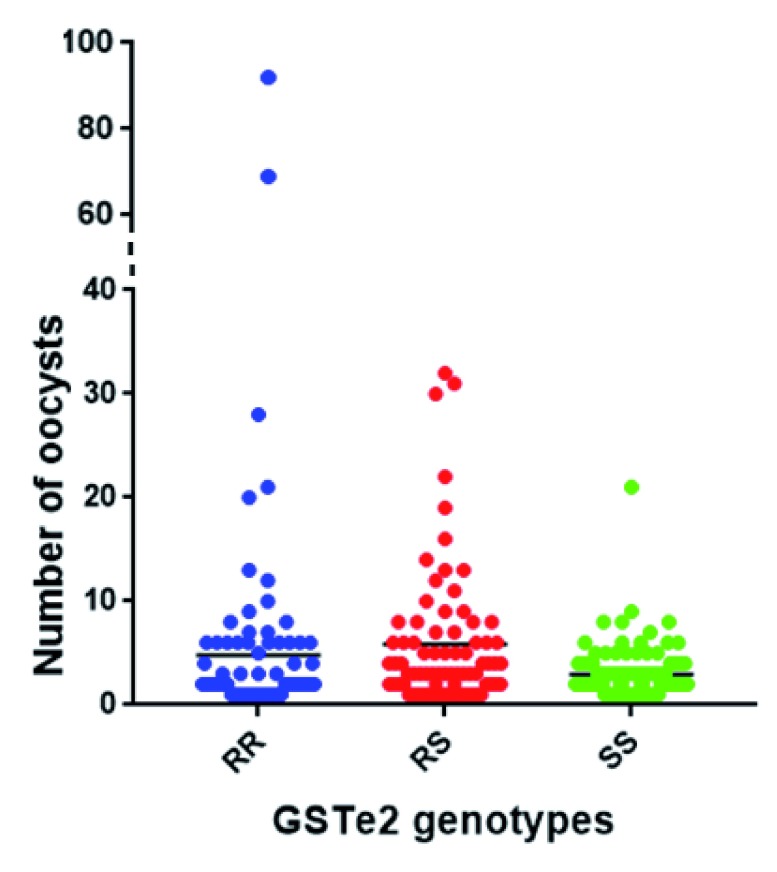
Infection intensity according to L119F-GSTe2 genotypes. Each dot represents a number of oocysts in a single midgut. It is possible that some dots are superposed. (SS: homozygous susceptible genotype, RS: heterozygous genotype, RR: homozygous resistant genotype).

A total of 218 and 185 mosquitoes was genotypes in Obout and Mibellon respectively. A non-uniform geographical distribution of L119F-GSTe2 resistance allele was observed with a higher frequency in Obout (65.93%) compared to Mibellon (25.95%). Distribution of *GSTe2* genotypes showed that homozygous resistant (RR: 50.40%) and heterozygous (RS:31.04%) were the most frequent in Obout, while SS (60%) was predominant in Mibellon ( Figure 1). For analysis of impact of *GSTe2* resistant allele on *An. funestus* vector competence, only experiments for which at least 20% of prevalence of infection was observed were considered ( Table 2). The frequency of the L119F-GSTe2 resistance allele was significantly higher in non-infected (55.88%) compared to infected (40.99%) mosquitoes (Fisher's exact test, P<0.0001). Heterozygous (RS:58.14%) and susceptible (SS:64.33%) mosquitoes were significantly more infected than their homozygous resistant (RR:40.14%) counterparts (Fisher's exact test, P: 0.0037 for RR vs SS; P<0.001 for RR vs SS; P:0329 for RS vs SS) ( Figure 2). As such, the odds ratio of being infected were significantly higher in RS and SS than in RR (OR: 2.07; 95%CI: 1.28-3.35 for RS vs RR; OR: 2.69, 95%CI:1.69-4.28 for SS vs RR; OR: 0.77, 95%CI: 0.48-1.24 for RS vs SS). The results of infection intensity were conflicting since mosquitoes bearing the resistant allele appeared to be much more permissive to oocyst infection ( Figure 3). Overall the number of oocyst found in a single midgut was significantly higher in heterozygous (Mean ± SEM: 5.80±0.77; Median: 3) and homozygous resistant genotypes (Mean ± SEM: 7.30±1.94; Median: 3) compared to susceptible mosquitoes (Mean ± SEM: 2.92±0.26; Median: 2) (Mann-Withney test, P:0.0323 for RR vs SS; P:0.0007 for RS vs SS; P:0.5011 for RR vs RS).

### *GSTe2* gene polymorphism and *Plasmodium* infection

A total of 34 sequences belonging to infected (N=18) and non-infected (N=16) mosquitoes were analyzed. The fragment length was 787 bp, spanning 3 exons and 2 introns and covering 92.6% of the full *An. funestus GSTe2* sequence ( AFUN015809-RA). Performing a BLASTn search in Vectorbase using the *An. funestus* sequence generated in this study revealed a very high-sequence homology (99.4%) with *An. funestus* full *GSTe2* gene sequence.

 Overall the diversity of the *GSTe2* fragment analyzed was low with only 15 (1.91%) polymorphic sites. All the 15 polymorphic sites were present in the non-infected group, while only 11 were found in infected group. This means that, nucleotide diversity was a bit higher in non-infected (π=0.005) compared to the infected (π=0.003) group. The mean numbers of nucleotide differences of the non-infected and infected groups were 3.667 and 2.510, respectively ( Table 3). However, no fixed mutation was observed between sequences of infected and non-infected mosquitoes.

**Table 3.** Summary of *GSTe2* sequence polymorphisms in infected and non infected mosquitoes.

		Conding region				Non-coding region			Whole sequence
		Exon1	Exon2	Exon3	All	Intron1	Intron2	All	
**Infected**	N seq	18	18	18	18	18	18	18	18
	N indiv	9	9	9	9	9	9	9	9
	Size	107	202	309	643	72	72	144	787
	Poly sites	2	1	2	6	4	1	5	11
	H	3	2	3	7	5	2	6	8
	Hd	0.307	0.209	0.529	0.791	0.484	0.336	0.562	0.797
	pi	0.003	0.001	0.002	0.002	0.009	0.005	0.007	0.003
	K	0.320	0.209	0.765	1.503	0.641	0.366	1.007	2.510
	Fu Li D	-0.552	0.667	0.885	0.577	-0.070	0.667	-0.359	0.164
	Fu Li F	-0.798	0.405	0.977	0.336	-1.008	0.708	-0.607	-0.121
	Tajima's D	-1.096	-0.529	0.769	-0.331	-1.347	0.488	-0.974	-0.786
**Non infected**	N seq	16	16	16	16	16	16	16	16
	N indiv	8	8	8	8	8	8	8	8
	Size	107	202	309	643	72	72	144	787
	Poly sites	1	2	3	6	3	6	9	15
	H	2	2	4	6	4	4	6	9
	Hd	0.325	0.125	0.692	0.842	0.517	0.442	0.675	0.883
	pi	0.003	0.001	0.003	0.003	0.008	0.021	0.014	0.005
	K	0.325	0.250	1.058	1.633	0.575	1.458	2.033	3.667
	Fu Li D	0.688	-1.915	-0.039	-0.706	-1.122	0.612	-0.051	-0.369
	Fu Li F	0.627	-2.060	0.117	-0.694	-1.262	0.306	-0.333	0.544
	Tajima's D	0.156	-1.498	0.495	-0.331	-1.055	-0.662	-0.919	-0.740

N seq: number of sequences; N indiv: number of individuals; Poly sites: polymorphic sites; H: haplotypes; Hd: haplotype diversity; Pi: nucleotide diversity; K: average number of nucleotide differences.

**Figure 4.**
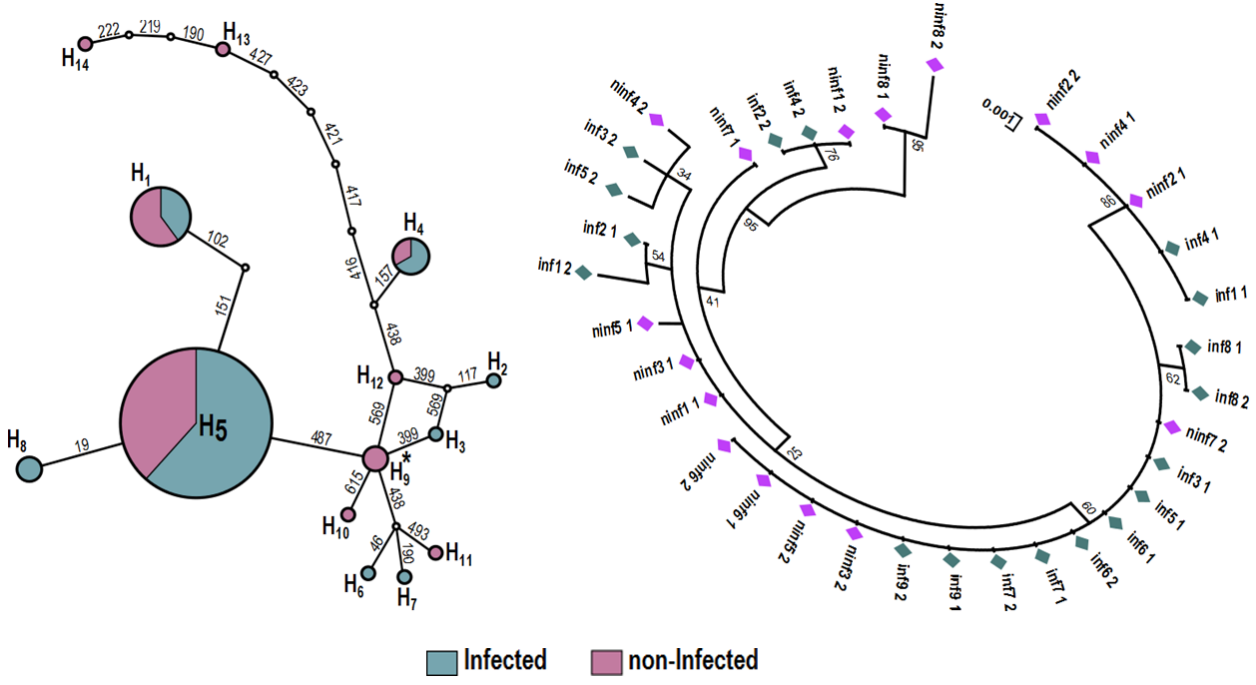
Haplotype network (left) and maximum likelihood phylogenetic tree (right) of *GSTe2* haplotypes in infected and non-infected *Anopheles funestus* mosquitoes.

 The substitutions defined 14 nucleotide haplotypes. Sequences of haplotypes have been deposited in GenBank under accession numbers MK439920 - MK439933. Haplotype diversity was slightly higher in the non-infected (0.883) compared to the infected group (0.797) ( Table 2). Six (42.86%) haplotypes appeared among non-infected specimens (Hap_9 - Hap_14), while 5 (35.71%) haplotypes were present in infected samples (Hap_2 - Hap_3, Hap_6 - Hap_8). Only 3 (21.43%) of the 14 haplotypes were shared between the two groups ( Figure 4). The major haplotype grouped 13 of 34 sequences (9 of RR and 4 of RS genotypes) and was shared between non-infected (61.54%) and infected (38.46%) samples. The haplotype network and the maximum likelihood phylogenetic tree did not reveal a clear segregation of haplotypes or individuals of these groups ( Figure 4). Moreover, Tajima's D, although negative were statistically non-significant (P>0.05) in all groups, suggesting no evidence of signature of selection ( Table 3).

## Discussion

In this study the distribution of the L119F-GSTe2 resistance allele and its impact on *P. falciparum* infection were assessed using mosquitoes originating from Obout and Mibellon (Cameroon). In these sites, *An. funestus* was by far the most abundant species collected, probably breeding in swamps formed by numerous rivers and lakes that promote the practice of fishing. This observation is in line with results of previous studies conducted in the same localities, during which high densities of resting blood fed *An. funestus* were collected in human dwellings. Using the Taqman technique, elevated *P. falciparum* infection rates reaching 23% were detected in this vector, thus underlying the major role it plays in malaria transmission ( Menze *et al*., 2018; Ndo *et al*., 2018; Ndo *et al*., 2016). All together these findings indicate that in these localities *An. funestus* remains an important malaria vector, despite large coverage of insecticide treated nets. It is possible that the high level of insecticide resistance detected in vector populations of the localities surveyed is one of the key factors responsible for the reduced efficacy of these vector control tools ( Menze *et al*., 2018; Ndo *et al*., 2018; Ndo *et al*., 2016).

A non-uniform geographical distribution of L119F-GSTe2 resistance allele was observed in the two *An. funestus* populations. The frequency of the allele was very high in Obout (65.93%) while it was low in Mibellon (29.95%). This result, particularly in Mibellon, did not correlate well with the high resistance to permethrin (mortality 48.88 ± 5.76%), deltamethrin (mortality was 38.34 ± 5.79%) and DDT (55.28 ± 8.28%) previously reported in this site, indicating that other mechanisms, such as overexpression of cytochrome P450s, could be involved ( Menze *et al*., 2018). Furthermore, this non-uniform distribution of the L119F-GSTe2 resistance allele also suggests that restriction of gene flow may exist between *An. funestus* populations in Cameroon, and/or that different selection pressures could have selected the resistance to insecticides in this species in both localities.

Whether the L119F-GSTe2 resistance allele impacts vector competence of *An. funestus* was investigated by comparing infection prevalence and intensity between mosquitoes belonging to RR, RS and SS genotypes, and by analyzing the polymorphisms in the *GSTe2* gene in infected and uninfected mosquitoes. We used the parafilm-glass feeding system for experimental infection of *An. funestus*. This technique can now be routinely used in this mosquito, similarly to *An. gambiae sl*, despite the observation that blood feeding rates remain low, likely because freshly field collected mosquitoes are not well adapted to feed through an artificial membrane ( Ndo *et al*., 2016). Similarly to the study of Ndo *et al.* ( Ndo *et al*., 2016), the two *An. funestus* populations used in this study also showed high susceptibility to natural *P. falciparum* gametocyte, a result that further confirms that this species is highly susceptible to the malaria parasite, hence its major role in the transmission of this disease in sub-Saharan African settings. By detecting higher overall infection rate in Mibellon, but higher infection intensity in Obout, our results did not allow us to clearly establish whether *An. funestus* populations from rainforests (Obout) are more or less competent to develop/transmit the malaria parasite than those from the humid savannah (Mibellon). Further studies using several mosquito populations from both ecoclimatic zones and fed on the same infectious blood are necessary. Sequence analysis did not reveal an association between *GSTe2* polymorphism and infection in field *An. funestus* mosquitoes, although sequencing more samples in the future could help to confirm this. On the other hand, infection prevalence and intensity were compared between mosquitoes of different genotypes, at oocyst level. It could have been also interesting to examine this impact at sporozoite level, which is the parasite stage transmitted to humans. However, the cumulative effect of the low number of mosquitoes fed on artificial membrane, the number dissected at day 7 and mosquito mortality before day 14, when sporozoites can be found in salivary glands, prevented us from carrying out this analysis. Nonetheless, analyzing such impact at oocyst level is reliable, as it has been shown that mosquito infectivity can be predicted with reasonable certainty from oocyst prevalence including in the cases of low intensity infections ( Stone *et al*., 2013). In general, the results obtained were contrasting. Both insecticide susceptible homozygote (SS) and heterozygote (RS) genotypes were significantly more susceptible to *P. falciparum* infection than the homozygote resistant (RR) genotype. In contrast results on the intensity of infection show that mosquitoes bearing the resistant L119F-GSTe2 allele (RR and RS genotypes) are more permissive to parasite infection than those with the SS genotype. This later observation suggest that GST-based metabolic resistance might interact with the immune system of the mosquito leading to a significant development of the parasite ( Ndiath *et al*., 2014; Rivero *et al*., 2010). Here, we suggest two possible explanations. The first is that overproduced GSTs might protect the parasites against the effects of reactive oxygen species (ROS), thus increasing the susceptibility of mosquitoes, by neutralising the oxidative response to the *Plasmodium*. ROS have been shown to have a role in insect innate immune responses as a potent pathogen-killing agent. For example, in *An. gambiae* Kumar *et al.* ( Kumar *et al*., 2003) demonstrated that ROS is involved in *P. berghei* melanotic encapsulation. However, Bahia *et al*. ( Bahia *et al*., 2013) reported that the interactions between *An. aquasalis* and *P. vivax* do not follow the model of ROS-induced parasite killing, indicating that the role of ROS in immune response to *Plasmodium* infection could vary according the *Anopheles*-parasite system. Future studies are therefore needed to investigate the role of ROS in *An. funestus* response to *P. falciparum* infection. The second explanation is that resource-based trade-offs could have affected mosquito immune-competency. For instance overproduction of detoxifying enzymes, such as esterases or GSTs, is likely to deplete the resource pool, limiting the vector's ability to mount an immune response, therefore favouring the development of the parasite ( Hall *et al*., 2009; Rivero *et al*., 2010). Considering this latter hypothesis, it could be likely that the low prevalence of infection in the RR genotype compared to the two others could have resulted in higher mortality before day 7 of individuals with a high load of infection. In fact mosquitoes of RR genotype may be less able to simultaneously maintain insecticide metabolic resistance and support development of a large amount of parasites, because of the limited host's energetic reserves. Likewise, Alout *et al.* ( Alout *et al*., 2016) also reported that *Plasmodium* infection reduced the survivorship of females in both resistant Acerkis and Kdrkis strains of *An. gambiae sl*.

 The effect of insecticide resistance on mosquito vector competence might differ according to the species and/or the resistance mechanism involved. In *An. gambiae*, Ndiath *et al.* ( Ndiath *et al*., 2014) also reported high parasite burden in RR and RS than SS genotypes. In another study, Alout *et al.* ( Alout *et al*., 2013) showed that the *kdr* resistant allele is associated with reduced parasite burden in infected individuals at the oocyst stage, when compared to the susceptible strain. In *An. funestus,* Lo *et al.* ( Lo & Coetzee, 2013) reported that pyrethroid resistant strain FUMOZ-R supported the lowest numbers of oocysts and sporozoites while the insecticide susceptible strain FUMOZ-BS produced highest sporozoite indices. In contrast, a recent study carried out by Tchouakui *et al.* ( Tchouakui *et al.*, 2019) showed that a GST-mediated metabolic resistance to insecticides is associated with high *Plasmodium* infection in field resistant *An. funestus*. Altogether, observations from the present and previous mentioned studies showed that insecticide resistance, including GSTe2-based metabolic-based resistance, may affect the development of the malaria parasite in mosquito vectors. Considering the importance of *An. funestus* in malaria transmission and the wide distribution of insecticide resistance in this mosquito, these results are of great concern for the epidemiology of malaria in sub-Saharan Africa. However, because several host external and internal factors could influence *Anopheles*- *Plasmodium* interactions, additional work needs to be done to further assess the impact of insecticide resistance on malaria transmission and epidemiology. To minimize the impact of confounding factors, such work should be carried out on mosquitoes sharing similar genetic backgrounds. Finally, future experiments should also explore the impact of *GSTe2* on *Plasmodium* infection at sporozoite level as it is this stage that is transmitted to human through mosquito bites.

## Data availability

### Underlying data

Open Science Framework: Exploring the impact of glutathione *S*-transferase (GST)-based metabolic resistance to insecticide on vector competence of *Anopheles funestus* for *Plasmodium falciparum.* https://doi.org/10.17605/OSF.IO/JMYWF ( Ndo, 2019)

This project contains the following underlying data:

- Gel1.tif (Representative gel image)
- Gel2.tif (Representative gel image)
- Gel3.tif (Representative gel image)
- Gel4.tif (Representative gel image)
- Gste2 gene sequence results.fas (Mosquito sampling and identification data)
- Raw data for infection and GSTe2 genotyping.xlsx (GSTe2 sequencing reads)

Data are available under the terms of the Creative Commons Zero "No rights reserved" data waiver (CC0 1.0 Public domain dedication).
