# Supplementary material for: Exploring the impact of glutathione  S-transferase (GST)-based metabolic resistance to insecticide on vector competence of Anopheles funestus for Plasmodium falciparum
Source: Wellcome Open Res. 2019 Oct 11;4:52. Originally published 2019 Mar 19. [Version 2] doi: 10.12688/wellcomeopenres.15061.2 (PMC6957023; doi:10.12688/wellcomeopenres.15061.2)
Supplement: Peer review history

# Peer review: Reviewer response for version 2

**DOI:** 10.21956/wellcomeopenres.16968.r37477

**Published:** 2020-01-29

**Authors:** Mamadou Ousmane Ndiath, Majidah Hamid-Adiamoh

**Affiliations:** 1 MRC Unit The Gambia at the London School of Hygiene and Tropical, Fajara, The Gambia

This paper entitled “ **Exploring the impact of glutathione *S*-transferase** **(GST)-based metabolic resistance to insecticide on vector** **competence of *Anopheles funestus *for *Plasmodium falciparum ***presents on the influence of glutathione S-transferase resistance on vector competence of *Anopheles funestus* in two sites in Cameroon. The authors found no association between *GSTe2 *variants and infection status of mosquitoes. It is a complex and difficult subject to quantify this relation because several factors and/or actions are involved in the vector-parasite relationship and each of them can negatively or positively affect the infection. From this point on and in view of the generalization of insecticide resistance, it becomes legitimate to ask this question. I am pleased to note that the authors have been able to appreciate the complexity of the issue and that, in the end, this is a preliminary study that should serve as a basis for moving forward, a point that they have developed well in the discussion.

 This version 2 of the manuscript is very elaborate as it takes into account comments and observations of the previous reviewers. See below my specific comments on the different sections of the manuscript.

**Abstract**

- It is advisable to state specifically that the study was carried out in *An. funestus* populations in Yaounde, Cameroon. A different result may be obtained from other regions outside this belt. “Here, we investigated whether glutathione S-transferase-based resistance could influence *Plasmodium falciparum* development in *Anopheles funestus”* .
- Use of GSTe2 gene and its polymorphism should be italicized.

**Methodology**

- Under mosquito sampling and identification, is there a specific reason why the authors were silent about the frequency of mosquito collection? Was it only a one-time point collection? The overall number of mosquitoes (615 in Obout and 670 in Mibellon) gave an indication that mosquitoes could have been collected multiple times from each site which was not indicated.
- The authors indicated that: “ *Progenies were pooled and reared to adulthood under standardized conditions*.” Which progenies were pooled? Is it progenies from the study sites or from different collection periods? It is important for the authors to emphasize this as the pooling strategies may be interpreted otherwise.

**Results**

***Anopheles funestus* infection**

- There is a difference between the values shown in the text and the corresponding table. Precisely in Obout, the infection rate on the text is 42.74% whereas in Table 2 it is 41.93%, the same is true for the intensity of infection, again in Obout: 7.44+-1.20 in the text and 7.57+-1.22 in the table. Which is the right number? Table or text?

**Distribution of L119F-GSTe2 resistance allele and *P. falciparum* infection**

- This section looks confusing to me! In the text it was indicated that **218** and 185 mosquitoes were genotyped in Obout and Mibellon respectively while in the title of Figure 1 we are talking about **128** mosquitoes in Obout. Could you revise the numbers?
- The percentages on their own don't mean anything, especially with the point I just mentioned before, so I recommend that you give the values of (n) each time you give percentages.

A typo error: “A total of 218 and 185 mosquitoes was **genotyped**" not genotypes in Obout and Mibellon respectively.

We confirm that we have read this submission and believe that we have an appropriate level of expertise to confirm that it is of an acceptable scientific standard, however we have significant reservations, as outlined above.

# Peer review: Reviewer response for version 2

**DOI:** 10.21956/wellcomeopenres.16968.r36739

**Published:** 2020-01-10

**Authors:** Dari F. Da

**Affiliations:** 1 Institut de Recherche en Sciences de la Santé, Bobo-Dioulasso, Burkina Faso

The manuscript in the version 2 included all comments and suggestion. The questions were clearly answered with more clarification. I give my approval for this article to be indexed.

I confirm that I have read this submission and believe that I have an appropriate level of expertise to confirm that it is of an acceptable scientific standard.

# Peer review: Reviewer response for version 1

**DOI:** 10.21956/wellcomeopenres.16431.r35232

**Published:** 2019-05-20

**Authors:** David P. Tchouassi

**Affiliations:** 1 International Centre of Insect Physiology and Ecology (icipe), Nairobi, Kenya

The authors investigated whether glutathione S-transferase-based resistance could influence *Plasmodium falciparum *development in *Anopheles funestus*. I commend the authors for the detailed piece of work. The experimental design and the findings are significant and address an important knowledge gap on the impact of insecticide resistance on relevant epidemiologic traits that can affect malaria control.

**Minor weaknesses**

 The authors found a lower infection rate in the mosquitoes expressing resistance allele but higher oocyst intensity in this genotype. I think challenging the mosquitoes with the target insecticide would help explain these discrepancies. besides, I have reservations as to whether 40% and 60% infection rates in the RR and SS genotypes are significant statistically.

 The authors use of very long sentences in especially in the discussion section. The thoughts could be communicated better in short simple sentences to improve on clarity. Kindly refer to the attached manuscript for comments and suggestions pertaining to typos or spelling mistakes

**Others**

**Abstract:**

 Background: Use of ‘interferes’ sounds as if it is a known fact; better to say ‘impact’.

 The authors found lower *Plasmodium *infection rate in the mosquitoes expressing resistance allele but higher oocyst intensity in this genotype. In light of this, I am not convinced about the authors being emphatic that GSTe2-based metabolic resistance increases the mosquito permissiveness to *Plasmodium* infection as stated in the conclusion. I think this sentence needs to be revised.

**Introduction:**

 Better to indicate the specific species in the *An. gambaie* sl where selection for resistance has been reported. This is a species complex and not sure such data exists for all members in this group. Also indicate clearly whether *An. funestus* refers to *An. funestus* s.s. 

 It is important to specify the mosquito species where the *GSTe2 *mutation has been detected. Does it occur in all malaria vectors?

**Materials and Methods**

 For the seasonal durations indicated, it may be helpful to indicate whether there may be year-to-year variations?

 The authors should indicate the specific vector species in *An. gambiae sl* found in Mibellon; not all species in this group are vectors.

 The sentence ‘ *Anopheles funestus *was found to actively transmit malaria in the locality, with an infection rate of up to 3.7% needs revision; a pathogen is transmitted but not a disease.

 Authors need to correct the description of RR, RS and SS on page 4 under L119F-GSTe2 mutation genotyping sub-title describing the DNA band sizes for each allele.

 Authors should avoid writing words in the contracted forms e.g. didn’t. this should be written in full and throughout the manuscript.

 It is better for the authors to replace the word ‘global’ to ‘overall’ as used in the results and discussion sections of the manuscript.

 Not sure about writing the all the p values to 4 dp.

I confirm that I have read this submission and believe that I have an appropriate level of expertise to confirm that it is of an acceptable scientific standard.

# Response

**Published:** 2019-10-03

**Authors:** Cyrille Ndo

**Affiliations:** OCEAC, Cameroon

**Abstract:**

**Reviewer's comment 1:  **Background: Use of ‘interferes’ sounds as if it is a known fact; better to say ‘impact’.

**Author's response 1:** correction done.

**Reviewer's comment 2:  **The authors found lower *Plasmodium *infection rate in the mosquitoes expressing resistance allele but higher oocyst intensity in this genotype. In light of this, I am not convinced about the authors being emphatic that GSTe2-based metabolic resistance increases the mosquito permissiveness to *Plasmodium* infection as stated in the conclusion. I think this sentence needs to be revised.

**Author's response 2:**  We added "possibly".

**Introduction:**

**Reviewer's comment 3:  **Better to indicate the specific species in the *An. gambaie* sl where selection for resistance has been reported. This is a species complex and not sure such data exists for all members in this group. Also indicate clearly whether *An. funestus* refers to *An. funestus* s.s. 

It is important to specify the mosquito species where the *GSTe2 *mutation has been detected. Does it occur in all malaria vectors?

**Author's response 3:** The specific species have been indicated.

**Materials and Methods**

**Reviewer's comment 4:  **For the seasonal durations indicated, it may be helpful to indicate whether there may be year-to-year variations?

**Author's response 4:** No, there is no year to year variation. We all know that seasons in a given region don't vary like that.

**Reviewer's comment 5:  **The authors should indicate the specific vector species in *An. gambiae sl* found in Mibellon; not all species in this group are vectors **.**

**Author's response 5:** The specific species has been indicated.

**Reviewer's comment 6:  **The sentence ‘ *Anopheles funestus *was found to actively transmit malaria in the locality, with an infection rate of up to 3.7% needs revision; a pathogen is transmitted but not a disease.

**Author's response 6:** We changed malaria by "plasmodium parasites"

**Reviewer's comment 7:  **Authors need to correct the description of RR, RS and SS on page 4 under L119F-GSTe2 mutation genotyping sub-title describing the DNA band sizes for each allele.

**Author's response 7:** We changed the sentece to " The size of the diagnostic band was  523 bp for homozygous resistant (RR)  and 312 bp for homozygous susceptible (RS), while heterozygous (SS) showed the two bands.

**Reviewer's comment 8:  **Authors should avoid writing words in the contracted forms e.g. didn’t. this should be written in full and throughout the manuscript.

**Author's response 8:** We removed all the contracted forms.

**Reviewer's comment 9:  **It is better for the authors to replace the word ‘global’ to ‘overall’ as used in the results and discussion sections of the manuscript.

**Author's response 9:** Global has been changed to overall.

**Reviewer's comment 10:  **Not sure about writing the all the p values to 4 dp.

**Author's response 10:** We don't think that this could change the sense of the results

# Peer review: Reviewer response for version 1

**DOI:** 10.21956/wellcomeopenres.16431.r35233

**Published:** 2019-05-08

**Authors:** Dari F. Da

**Affiliations:** 1 Institut de Recherche en Sciences de la Santé, Bobo-Dioulasso, Burkina Faso

The manuscript (Exploring the impact of glutathione S-transferase (GST)-based metabolic resistance to insecticide on vector competence of *Anopheles funestus* for *Plasmodium falciparum*) addresses an important question in the field epidemiology of malaria transmission. Fully elucidating major interactive parameters that are regulating Plasmodium transmission in malaria endemic areas is essential in the control of the disease. The content of this study, although superficial and required to be reformulated, appears as an important contribution in terms of the patterns between mosquito insecticide resistance and their vector capacity. Consistent design was presented with the appropriate methodology to achieve the objective mentioned. The global results were well described but some details remain to be completed (see comments attached in the pdf version of the manuscript). Some comparative analysis must be completed to clearly elucidate the relationship between the main parameters taking account some important variables. Because of this, superficial analysis in this version the discussion was likely based in large part on the previous studies. **

I confirm that I have read this submission and believe that I have an appropriate level of expertise to confirm that it is of an acceptable scientific standard, however I have significant reservations, as outlined above.

# Response

**Published:** 2019-10-03

**Authors:** Cyrille Ndo

**Affiliations:** OCEAC, Cameroon

**Reviewer's comment** **1.** change "mosquito" to "An. funestus"

**Author's response 1:** we changed ''mosquitoes'' to ''An. funestus mosquitoes''

**Reviewer's comment** **2. **This last paragraph, essential to clearly highlight your problematic is unfortunately formulated with only 2 long sentences rendering confused hypothesis and objective. It requires to be rewritten with more details to fully express your scientific question.

**Author's response 2:**  The paragraph has been rewritten.

**Reviewer's comment** **3**. You clearly provided some details about Mibellon, one of your study sites, but the insecticide-resistance history of the major vectors in this locality is not mentioned here. Because your objective focus to explore the eventual impact of the genotype of the vectors in relation with their competence, it was expected to find the the mosquito insecticide background motivating your site choice. What comment could you provide here?

**Author's response 3:** You are right and we therefore added this information as follow:

"Both vectors have developed high levels of resistance to pyrethroids (deltamethrin and permethrin) and organochlorides (DDT) and moderate resistance to carbamate (bendiocarb) ( Menze *et al*., 2018)."

**Reviewer's comment** **4.** 30 out of 400 An funestus were used for gene sequencing (so less than 10% of your mosquito population). The 400 Anopheles (F1 generation) originating from more than thousand of field mothers (615+670) present evidently a various genetic background. The sequencing results of 30 out of 400 An funestus (a sampling in a sample) seem too small to be representative and could provide inappropriate tendencies. In the absence of reasonable proofs about this choice, an alternative solution would consist of sequencing more anopheles genes to increase your sample size if the DNA is always available.

**Author's response 4:** although analyzing 30 sequences of individuals out of 400 could seem small, it is insufficient provide appropriate tendances. In fact number of studies published in peer reviewed journals have generated very robust results by analyzing similar sample size or less per population. We can cite for example:

- Ndo *et al. *2010 (malaria journal): Population genetic structure of the malaria vector Anopheles nili in sub-Saharan Africa (5 to 10 individuals analyzed per population)
- Menze *et al.* 2016 (Plos one): Multiple Insecticide Resistance in the Malaria Vector Anopheles funestus from Northern Cameroon Is Mediated by Metabolic Resistance Alongside Potential Target Site Insensitivity Mutations (5 dead and 5 alive sequenzed)
- Rireveron *et al.* 2014 (Genome biology): A single mutation in the GSTe2 gene allows tracking of metabolically based insecticide resistance in a major malaria vector. (5-12 individuals analyzed per population)
- Weedall *et al*, 2019 (Sciences translational medicine): A cytochrome P450 allele confers pyrethroid resistance on a major African malaria vector, reducing insecticide-treated bednet efficacy. (15 individuals analyzed in each of the 10 countries across Africa).

**Reviewer's comment** **5**. It is important to give the precision on the calculated mean:  arithmetic or geometric? Mean estimated in the dissected population of Anopheles or only in those infected by Plasmodium oocyst? Depending on the study objective, evaluate the impact of intervention  (vaccine, drug, ...) or just understand some biological mechanism in the parasite transmission the estimated oocyst mean and the mosquito population considered could be different.

**Author's response 5:** we now gave the precision in the text that it was arithmetic mean.

**Reviewer's comment** **6.** If you have no conviction that several species were found in the locality, "An. gambiae sl" is more appropriated "than An. gambiae complex".

**Author's response 6:** We changed An. gambiae complex to An. gambiae sl.

**Reviewer's comment** **7.** In the paragraph highlighted (however ... P:0.0003) it is understood than you try to compare the mosquito infection level between the 2 sites (Mibellon and Obout). This comparison has no sense for the reason that different human volunteers with of course various gametocytemia were used to perform the experiment. Gametocyte density, an important parameter in the mosquito infection could maybe help to argue this statement.

**Author's response 7:** We agree with the reviewer that use of different gametocyte carrier could lead to different infection parameters. The paragraph has been modified as follows:

Overall, the blood feeding rate of *An. funestus* through the artificial parafilm membrane was low compared to that of the *An. coluzzii* Ngousso strain used as control (80.42% - 100%) and didn't exceed 40% in all the cases (min - max: 1% - 38%). *Anopheles funestus* of both sites showed high susceptibility to natural *P. falciparum* isolates with 72.73% (8/11) and 77.78% (7/9) experiments yielding at least one infected mosquito in Obout and Mibellon, respectively. The overall infection rate was 69.73% Mibellon and  42.74% in Obout. By contrast infection intensity represented by mean and median oocyst load in midgut was moderate in Obout (mean: 7.44±1.20; median: 4) and low in  Mibellon (mean: 2.88±0.18; median: 2)(Table 2).

**Reviewer's comment** **8.** This last sentence (When the data ... significant) likely describes the opposite of what is observed in the table: From one experience to another and for each significant variations seem evident for both prevalence and oocyst intensity. Could you clearly include the comparative statistic tests performed to support your assertion?

**Author's response 8:** After having modified the paragraph above, we found necessary to remove the last sentence to make more sense

**Reviewer's comment** **9.** For which reasons only experiments with 20% infection prevalence were considered for genotypic analysis?

**Author's response 9:** We use only experiments with at least 20% to be sure that gametocyte (blood) was really infective, but more importantly, this allowed us to have enough individual infected or non infected of different genotypes

**Reviewer's comment** **10. **In this paragraph (The  frequency ...  RR vs RS), the authors must account for the origin of the mosquitoes (site) and the infectious doses (gametocytemia) in their analyses of the effects of resistance on parasite prevalence and intensity in mosquitoes. Because there are differences in resistance frequencies among villages (Fig 1), this observation has to be considered in the following statistical analyses of mosquito infection.

Such analysis will provide more information about what is qualified "contrasting results" and the discussion could be reformulated.

**Author's response 10:** In this paragraph, the analysis is not done according to the origin of mosquitoes but according to the genotype, irrespective of the origin. Since all the genotypes from a single experiment received the same infectious blood meal (same gametocytemia) and are all include in the analysis, we think that observations done are meaningful.
